# Hypothyroidism and metabolic cardiovascular disease

**DOI:** 10.3389/fendo.2024.1408684

**Published:** 2024-06-03

**Authors:** Armando Patrizio, Silvia Martina Ferrari, Giusy Elia, Francesca Ragusa, Eugenia Balestri, Chiara Botrini, Licia Rugani, Valeria Mazzi, Alessandro Antonelli, Poupak Fallahi, Salvatore Benvenga

**Affiliations:** ^1^ Department of Emergency Medicine, Azienda Ospedaliero-Universitaria Pisana, Pisa, Italy; ^2^ Department of Clinical and Experimental Medicine, University of Pisa, Pisa, Italy; ^3^ Department of Surgical, Medical and Molecular Pathology and Critical Area, University of Pisa, Pisa, Italy; ^4^ Department of Translational Research and New Technologies in Medicine and Surgery, University of Pisa, Pisa, Italy; ^5^ Department of Clinical and Experimental Medicine-Endocrinology, University of Messina, Messina, Italy; ^6^ Master Program on Childhood, Adolescent and Women’s Endocrine Health, University of Messina, Messina, Italy; ^7^ Interdepartmental Program of Molecular & Clinical Endocrinology and Women’s Endocrine Health, University Hospital Policlinico “G. Martino”, Messina, Italy

**Keywords:** overt hypothyroidism, subclinical hypothyroidism, cardiovascular disease, levothyroxine, TSH, thyroid hormones, LDL

## Abstract

Cardiovascular disease (CVD) remains the leading cause of death worldwide, representing a major health issue of social and economic relevance. Both hyperthyroidism and hypothyroidism are very common in the adult population, and both disorders may contribute to the onset and progression of CVD. After a brief description of the role of thyroid hormones (THs) on the physiology of the cardiovascular system and the potential mechanism that links THs alterations with changes in cardiac function, blood pressure, endothelial function, and lipid levels, we review updated data about the clinical impact of overt hypothyroidism (OH) and subclinical hypothyroidism (SCH) on CV risk, CVD, and mortality. Furthermore, we summarize the current evidence for treating SCH with levothyroxine (L-T4). Several guidelines of distinguished endocrine societies recommend treatment for SCH with TSH higher than 10 mIU/L, where the benefit of L-T4 therapy is more evident for younger people, but still controversial in those aged over 65 years. Based on current knowledge, more research efforts are needed to better address the clinical management of CV risk and CVD in the elderly affected by SCH.

## 1 Introduction

Cardiovascular diseases (CVDs) represent not only the leading cause of death worldwide, but also the most important cause of morbidity, with high impact in terms of cost to the healthcare systems (1). For this reason, it is necessary to improve the management and preventive strategies in order to reduce the CVD burden.

Thyroid diseases are the most frequent endocrine disorders, affecting more than 10% of the general adult population (2, 3). Thyroid hormones (THs), viz. free triiodothyronine (fT3) and free thyroxine (fT4), play an important role in the human homeostasis, since they regulate growth, development, and energy metabolism, starting from the intrauterine life. TH receptors (TRs) are ubiquitous and, therefore, they are also present in the myocardium and vascular endothelial tissues (4). The significant clinical impact of overt thyroid dysfunction on the cardiovascular (CV) system is well documented in patients with overt hypothyroidism (OH) and hyperthyroidism, while it is more debated for their milder, subclinical forms. The absence of high-quality evidence for such subclinical forms of thyroid dysfunction has led to controversy over their optimal management.

OH is defined by elevated serum levels of thyrotropin (TSH) co-existing with low circulating levels of THs, a disorder that affects 0.2% to 2% of nonpregnant adults (5, 6). Subclinical hypothyroidism (SCH) is diagnosed when fT4 is within the normal range, while TSH is beyond the upper normal limit (>4.0 mIU/L). The main causes of OH and SCH are summarized in **Table 1**, with Hashimoto’s thyroiditis (HT) being the most common worldwide (7). Both OH and SCH are associated with several CVDs and increased risk of having classical CV risk factors, such as hypertension, dyslipidemia, and increased carotid intima media thickness (CMT) (4).

**Table 1:** Causes of acquired hypothyroidism and subclinical hypothyroidism.

Autoimmune diseases	Chronic lymphocytic thyroiditis, Hashimoto’s thyroiditis, blocking TRAbs, post-partum thyroiditis, and painless thyroiditis
Infectious	Thyroid abscess and subacute thyroiditis
Thyroid infiltration diseases	Amyloidosis, hemochromatosis, acquired immunodeficiency syndrome, sarcoidosis, Riedel’s thyroiditis, cystinosis, scleroderma, and secondary tumors
Iatrogenic	Drugs: Antithyroid drugs, lithium, amiodarone, tyrosine kinase inhibitors, immune checkpoint inhibitors, interferon therapy, radiographic contrast agents, sulfonamides, bexarotene (retinoid X receptor agonist), and dopamine
	Postablative thyroiditis due to 131I, surgery, or therapeutic irradiation for nonthyroidal malignancy
Iodine deficiency	Endemic goiter
Central hypothyroidism	Pituitary origin (secondary), hypothalamic disorders (tertiary), and severe illness

After a brief description of the physiologic mechanism of THs on the CV system, the aims of this review are (i) to summarize the impact of OH and SCH on the development of CV risk factors, CVD and, mortality and (ii) to address the current knowledge about the effects of their treatment with levothyroxine (L-T4). With this scope, we have performed a comprehensive review of the English literature, published since 2000 and extracted from the PubMed/Medline database, on studies about the relationship of thyroid function and treatment with the CV system and diseases. We included original articles, reviews, viewpoints, commentaries, case series, and case reports. The search terms, used in combination, included “hypothyroidism”, “subclinical hypothyroidism”, “cardiovascular disease”, “cardiovascular system”, “levothyroxine”, “TSH”, and “heart and thyroid hormones”.

## 2 Physiology of thyroid hormones on the cardiovascular system

THs exert their effects through genomic (delayed) and non-genomic (rapid) pathways. The genomic action is mediated by nuclear TRs, which are of two isoforms (TRalfa and TRbeta). T3 has higher affinity (approximately 20–30 times) than T4 for TRs (8). These TRs upregulate or downregulate the expression of several genes. The targeted genes are involved in several actions, such as the contractile apparatus of the cardiomyocyte, in the regulations of calcium ion kinetics, as well as in gluconeogenesis and lipogenesis (9). On the other hand, the non-genomic effects act on the autonomous nervous system and renin–angiotensin–aldosterone system, which, in turn, have an impact on both the heart and vessel functions.

### 2.1 Action on the heart

Inside the cardiomyocyte, THs regulate the synthesis of a number of proteins, including myosin heavy chains (MHCs), the sarcoplasmic reticulum calcium adenosine triphosphatase (SERCA2) and its inhibiting counterpart phospho-lamban (PLB), and b1-adrenergic receptors; all these proteins contribute to the lusitropic, dromotropic, and chronotropic effects of THs (10). The non-genomic effects are TR-independent, and largely occur at the plasma membrane, with effects on the regulation of ion channel activity (Ca2+ ATPase, Na+/K+/ATPase) and transport of glucose and amino acids (11–13). THs seem to protect myocardial cells from oxidative stress by stimulating the expression of specific microRNAs, promoting the production of nitric oxide (NO) and decreasing the production of reactive oxygen species (ROS) (14–16).

The endothelial cells are also susceptible to THs. In fact, THs regulate ion channel activation (Na+, K+, Ca2+) and specific signal transduction pathways. Activation of phosphatidylinositol 3-kinase and serine/threonine protein kinase downstream causes the production of endothelial NO, leading to a reduction in systemic vascular resistance (17, 18).

Finally, lipid metabolism and insulin signaling are also regulated by THs especially in the liver, where THs stimulate the expression of low-density lipoprotein cholesterol (LDL) receptors and the activity of lipoprotein lipase (19).

## 3 Cardiovascular manifestations of overt and subclinical hypothyroidism

A recent retrospective cohort study from UK analyzed almost 900,000 repeated TSH measurements, in a well-balanced and highly powered population of 162,369 patients with hypothyroidism, comparing them with the reference TSH interval of 2–2.5 mIU/L: the study showed that the risk of ischemic heart disease and heart failure increased at high TSH concentrations (>10 mIU/L) [HR 1.18 (95% CI 1.02 to 1.38; *p* = 0.03) and 1.42 (95% CI 1.21 to 1.67; *p* < 0.001), respectively]. A protective effect for heart failure was seen at low TSH concentrations [HR 0.79 (95% CI 0.64 to 0.99; *p* = 0.04) for TSH <0.1 mIU/L and 0.76 (95% CI 0.62 to 0.92; *p* = 0.006) for 0.1–0.4 mIU/L]. Interestingly, fragility fractures were more common at highest TSH concentrations (>10 mIU/L) [HR 1.15 (95% CI 1.01 to 1.31; *p* = 0.03]. These results have been observed also for mortality, which was increased for TSH categories >4 mIU/L (HR 1.29, 95% CI 1.22 to 1.36; *p* < 0.001), raising concerns about the benign course of SCH and underlying the need for stricter monitoring in this setting (20). Considering the elevated numbers of the analyzed data, this study represents one of the hardest evidence of the negative impact of higher TSH values on CV outcomes and all-cause mortality. These results have also been confirmed in different populations all around the world such as USA (21) and Asia (22, 23). Furthermore, several other prospective cohort studies demonstrated that SCH is associated with a higher burden of CVD and death especially among those with TSH higher than 10 mIU/L (24–28).

OH can impact the CV system in several aspects, such as by determining reduced stroke volume and cardiac contractility, and increasing peripheral vascular resistance with subsequent hypertension and diastolic dysfunction (13). Patients with OH can also display typical electrocardiographic patterns such as sinus bradycardia, prolonged QT interval, and atrioventricular block (29). OH worsens modifiable CV risk factors, inducing hypercholesterolemia, increased carotid intima-media thickness (CIMT), and reduced endothelial NO mediated relaxation (30). Low levels of THs decrease lipoprotein lipase activity in hepatic and adipose tissue, with subsequent impairment of the cholesterol metabolism that shifts in favor of increasing the circulating levels of total cholesterol and LDL-c levels (31, 32). Taken together, all these metabolic and vascular changes contribute to the inflammation, oxidative stress, and endothelial dysfunction of the vessel walls, which therefore become more vulnerable to the development and the progression of atherosclerosis (33).

The most frequent cardiac impairment found among people affected by SCH is diastolic dysfunction due to altered ventricular filling and relaxation (33), unmasked by reduced exercise tolerance (34). The Whickham survey cohort revealed higher systolic and diastolic blood pressures and hypercholesterolemia in patients with SCH than in euthyroid people (24). However, data from population studies about the association between SCH and either CV morbidity or mortality are not always concordant. For instance, the EPIC-Norfolk study showed that although people with SCH carried a worse CV risk profile, they did not display a higher rate of coronary heart disease (CHD) and all-cause mortality during the 10 years of follow-up (35).

## 4 The role of therapy with levothyroxine

Thyroid dysfunctions are very common among people admitted for CVD, with SCH being the most common disorders (36). In fact, the recommendations by the American College of Cardiology/American Heart Association and the European Society of Cardiology suggest to screen for thyroid diseases all patients affected by atrial fibrillation (AF), heart failure (HF), and CHD to detect and manage these potential secondary causes of CVD (37, 38).

L-T4 is the cornerstone of hypothyroidism treatment. The joint consensus of the American Association of Clinical Endocrinologists (AACE) and the American Thyroid Association (ATA) recommends L-T4 therapy for OH and for SCH when TSH levels are higher than 10 mIU/L, with an initial dose of 20–25 µg/day for older people (>80 years) (39). L-T4 therapy seems to improve left ventricular function, contractility, endothelial function, lipemic profile, and cardiac mitochondrial function, thus at least partially reversing the pathological impact of OH and SCH on the CV system (40).

A review showed a CV benefit of L-T4 treatment in patients with SCH (TSH > 10 mIU/L and thyroid autoantibody-positive), since CV events decreased and quality of life improved (41). Based on a meta-analysis of 16 studies with SCH, hormonal therapy ameliorated several surrogates of endothelial function and arterial stiffness [total cholesterol and LDL-c level, C-reactive protein (CRP), brain natriuretic peptide (BNP), and flow-mediated dilatation (FMD)], suggesting a potential positive impact of L-T4 on the CV system (42). The small experimental study of Niknam et al. supports these results, demonstrating an increase in FMD in young patients with SCH compared to controls (43).

In addition, a meta-analysis of 12 clinical trials, where SCH patients were treated with L-T4, demonstrated an improved profile of atherosclerosis and CV risk, secondary to significant reduction of CIMT values and lipid levels (44).

Taken together, these biochemical and clinical evidence support L-T4 treatment to both prevent CVD and provide significant CV benefit among patients affected by SCH.

However, above all, an age-related difference in CVD and mortality outcomes secondary to L-T4 in SCH emerges (45). For instance, in a meta-analysis of seven studies and more than 20,000 patients, people aged less than 70 years and on hormonal replacement therapy were associated with reduced overall [relative risk (RR) 0.50, 95% CI 0.29–0.85, *p* = 0.011] and CV mortality (RR 0.54, 95% CI 0.37–0.80, *p* = 0.002), with respect to older patients, where no statistically significant association was found. In contrast, another study from Denmark confirmed these benefits also in treated patients older than 65 years (HR 0.84, 95% CI 0.75–0.94, *p* = 0.002) (46). In addition, one analysis from the United Kingdom, based on the General Practitioner Research Database (GPRD), showed that ischemic heart disease events were less frequent among patients aged 40 to 70 years with SCH and on L-T4 [68 events in 1,634 treated patients (4.2%) versus 97 events in 1,459 untreated patients (6.6%); HR 0.61, 95% CI 0.39–0.95], than among treated individuals over age 70 years [104 events in 819 patients on L-T4 (12.7%) versus 88 events in 823 untreated patients (10.7%), HR 0.99, 95% CI 0.59–1.33] (47). Finally, in a retrospective study from Denmark including 12,212 people affected by SCH, no difference in the risk of myocardial infarction or CV mortality was detected between L-T4-treated and untreated patients, even though those younger than 65 years showed reduced overall mortality compared with those untreated (incidence rate 4.2 versus 6.5 per 1,000 person-years, incidence rate ratio 0.63, 95% CI 0.40–0.99) (48).

Specifically, in a placebo-controlled randomized clinical trial (RCT) of L-T4 or placebo for an average time of 18 months in 185 patients older than 65 years with TSH 4.6 to 19.99 mIU/L, 18 months of hormonal therapy did not significantly improve systolic function. Of note, in this study, the involved older adults had mild SCH (few patients with TSH ≥10 mIU/L, and mean achieved TSH values were 3.55 and 5.29 mIU/L in the treatment and placebo arms, respectively) (49). A small case–control study even suggested that L-T4 therapy in people aged more than 65 years and with mild SCH (TSH between 4.2 and 10 mIU/L) is associated with an increased overall mortality (HR 1.19, 95% CI 1.03–1.38) (50). In a retrospective Danish study of 1,192 people with established CVD and SCH (mean age 73.6 years), there was no difference in the risk of all-cause mortality or major adverse CV events (myocardial infarction, CV mortality, stroke, or all-cause hospital admissions) between patients treated or not treated with L-T4 (51). A double-blind RCT from the United Kingdom showed no improvement after 52 weeks of hormonal replacement therapy among critically ill patients with SCH and acute myocardial infarction (52).

Therefore, although treatment of SCH is still debated, L-T4 therapy should be administered to younger patients. More and longer RCT are certainly needed to assess the clinical safety and benefit of L-T4 therapy in terms of CVD outcomes among older adults (>65–70 years) affected by this thyroid dysfunction, bearing in mind that association between increased mortality and highest TSH remained in both the younger and older subgroups, after age-stratified analysis (20).

Last but not least, undertreated primary hypothyroidism is observed in ~20% of primary hypothyroid patients treated with tablet L-T4 (53). In approximately one-third of such patients, the cause of undertreatment is oral ingestion of different medications that interfere with the intestinal absorption of L-T4, such as proton pumps inhibitors (PPIs), ferrous sulfate, sucralfate, raloxifene, calcium and iron supplements, phosphate binders, and aluminum-containing antacids or bile sequestrants (54, 55). In a retrospective study, 114 patients with primary hypothyroidism under L-T4 therapy were enrolled; they did not receive those medications for 2 years minimum (baseline), while they received them for another 2 years minimum (exposure). The study evaluated TSH serum levels (with proportions of serum levels > 4.12 mIU/L) and rate of complications, such as pre-existing or *de novo* onset of any of metabolic syndrome, impaired fasting glycemia, diabetes mellitus, dyslipidemia, hypertension, CHD, and CVD. It was shown that the average TSH level during exposure was 3.44 ± 4.08 mIU/L (median 2.10) in the 76 patients who experienced complications, and 1.58 ± 1.98 (median 1.28) in the 38 patients who did not, with the respective baseline levels being 1.18 ± 1.44 (median 0.86) and 1.43 ± 1.09 mIU/L (median 0.97). For this reason, the interference on the intestinal absorption of tablet L-T4 caused by certain medications leads TSH to move from one class of metabolic/CV risk toward classes of a higher risk (56).

More recently, pharmaceutical research has led to the availability of new formulations of L-T4, other than the classic tablet (57–67).

## 5 Conclusions

Several clinical and laboratory data give compelling evidence on the role of THs in the normal physiology of the CV system and on the negative cardiac impact deriving from even subtle impairment in thyroid function. OH and SCH are widespread disorders, which are often detected in people with high CV risk or established CVD. Because of the meaningful relationship between reduced thyroid function, CVD, and mortality, many efforts have been made to establish the best management with L-T4 therapy in these populations. While the benefit of the treatment of OH and younger patients with SCH has more solid evidence, for people with SCH aged over 65–70 years, it still demands further investigations. Therefore, long-term and wider clinical trials should be performed to better address this clinical issue and reduce CVD incidence and CV mortality.

## Author contributions

AP: Writing – review & editing, Writing – original draft, Formal Analysis, Data curation. SMF: Writing – review & editing, Writing – original draft, Formal Analysis, Data curation. GE: Writing – review & editing. FR: Writing – review & editing. EB: Writing – review & editing. CB: Writing – review & editing. LR: Writing – review & editing. VM: Writing – review & editing. AA: Writing – review & editing, Writing – original draft, Formal Analysis, Data curation. PF: Writing – review & editing, Writing – original draft, Formal Analysis, Data curation. SB: Writing – review & editing, Writing – original draft, Formal Analysis, Data curation.
